# Complex Dynamics in a Memcapacitor-Based Circuit

**DOI:** 10.3390/e21020188

**Published:** 2019-02-16

**Authors:** Fang Yuan, Yuxia Li, Guangyi Wang, Gang Dou, Guanrong Chen

**Affiliations:** 1 College of Electrical Engineering and Automation, Shandong University of Science and Technology, Qingdao 266590, China; 2 Institute of Modern Circuits and Intelligent Information, Hangzhou Dianzi University, Hangzhou 310018, China; 3 Department of Electronic Engineering, City University Hong Kong, Hong Kong 999077, China

**Keywords:** chaos, memcapacitor, extreme multistability, circuit emulator, complex dynamics

## Abstract

In this paper, a new memcapacitor model and its corresponding circuit emulator are proposed, based on which, a chaotic oscillator is designed and the system dynamic characteristics are investigated, both analytically and experimentally. Extreme multistability and coexisting attractors are observed in this complex system. The basins of attraction, multistability, bifurcations, Lyapunov exponents, and initial-condition-triggered similar bifurcation are analyzed. Finally, the memcapacitor-based chaotic oscillator is realized via circuit implementation with experimental results presented.

## 1. Introduction

Memcapacitor is a type of memory device composed of a memristor and meminductor [1], which emerged after the realization of a real memristor prototype [2]. A memcapacitor is actually a nonlinear capacitor with instantaneous responses depending on the internal states and the input signals. Although several possible realizations of the memcapacitor were attempted, e.g., using a micro-electro-mechanical system [2], ionic transport [3], or electronic effect [4], a memcapacitor is not yet available commercially in any form. Therefore, building functional analog memcapacitor models and emulators for computer simulations and laboratory experiments has become urgent, attracting immense interest from both academia and industry.

There have been many papers about the memristor [5,6,7,8,9]. In [6], a novel complex Lorenz system with a flux-controlled memristor is introduced and investigated. An active controller is designed to achieve modified projective synchronization (MPS) based on Lyapunov stability theory. In [7], a new memristor-based hyperchaotic complex Lu system is investigated, where an adaptive controller and a parameter estimator are proposed to realize complex generalized synchronization. Furthermore, the complex dynamics of fractional-order and diode bridge-based memristive circuits are studied in [8] and [9],, respectively. Compared with the reports concerning memristors, references of memcapacitors are relatively fewer. Existing research involves designing a memcapacitor SPICE (Simulation program with integrated circuit emphasis) simulator [10,11,12] and mutator that can transform a memristor to a memcapacitor [13,14]. In [15], the boundary dynamics of a charge-controlled memcapacitor is investigated, where Joglekar’s window function is used to describe the nonlinearities of memcapacitor’s boundaries. In [16], a mathematical memcapacitor model is introduced and a memcapacitor oscillator is designed, with theoretical and experimental analyses on their basic dynamic characteristics given. In [17], a floating emulator circuit is built, using common off-the-shelf active devices, to mimic the dynamic behaviors of flux-coupled memcapacitors.

Memcapacitors are also employed to construct chaotic circuits. A smooth-curve memcapacitor model and a memcapacitive chaotic circuit are presented in [18], where different kinds of coexisting attractors are shown and their corresponding conditions are given. In [19], a Hewlett–Packard memristor model and charge-controlled memcapacitor model are presented, based on which, a new chaotic oscillator is designed to explore characteristics of memristors and memcapacitors in nonlinear circuits. In [20], a chaotic oscillator composed of a meminductor and a memcapacitor is proposed, and in [21] a chaotic memcapacitor-based oscillator with two unstable equilibrium points are studied, along with its fractional form, for potential engineering applications. In the latest study [22], a buckled membrane is used as the plate of a capacitor with memory to realize the function of a memcapacitor.

Along the same line as the above extensive investigations, in this paper, a new memcapacitor model is proposed and its corresponding emulator is designed. The instantaneous responses of the emulator to internal states and input signals are investigated experimentally by applying different voltage excitations. Furthermore, a memcapacitor-based chaotic oscillator is constructed. The system dynamic behaviors are analyzed theoretically, including coexisting attractors, basins of attraction, extreme multistability, and so on. Compared with the previously published papers, a memcapacitor with absolute value relation is first proposed and the corresponding emulator can be directly used in application circuits. Besides, we further investigate the similar bifurcation structures triggered by different initial conditions, which has not been reported in memcapacitive systems yet. Finally, the proposed oscillator is realized via analog circuits, verified by laboratory experiments.

## 2. The Memcapacitor Model and Its Emulator 

The concept of memcapacitor was presented by Chua et al. [1], where a charge-controlled memcapacitor is described using:

(1) $$\{\begin{array}{l} v_{c}\left(t\right)=C^{-1}\left(\sigma_{M}\left(t\right)\right)q_{M}\left(t\right)\\ {\dot{\sigma }}_{M}\left(t\right)=q_{M}\left(t\right) \end{array}$$

where *v_c_*(*t*) is the voltage across the memcapacitor and *C*^−1^ is the inverse memcapacitance. The symbol *q_M_*(*t*) is the charge going through the memcapacitor at time *t*, and *σ_M_*(*t*) is the integral of *q_M_*(*t*). 

To describe the proposed charge-controlled memcapacitor model, Equation (2) is used, where the memcapacitance depends on the device charge and is changing nonlinearly. Since the meminductor has not been fabricated, in this paper we assume the inverse memcapacitance to be precisely defined as *C*^−1^ = *a* + *b*|*σ_M_*(*t*)|, so as to obtain:

(2) $$\{\begin{array}{l} v_{c}\left(t\right)=\left(a+b\left|\sigma_{M}\left(t\right)\right|\right)q_{M}\left(t\right)\\ {\dot{\sigma }}_{M}\left(t\right)=q_{M}\left(t\right) \end{array}$$

**Figure 1:**
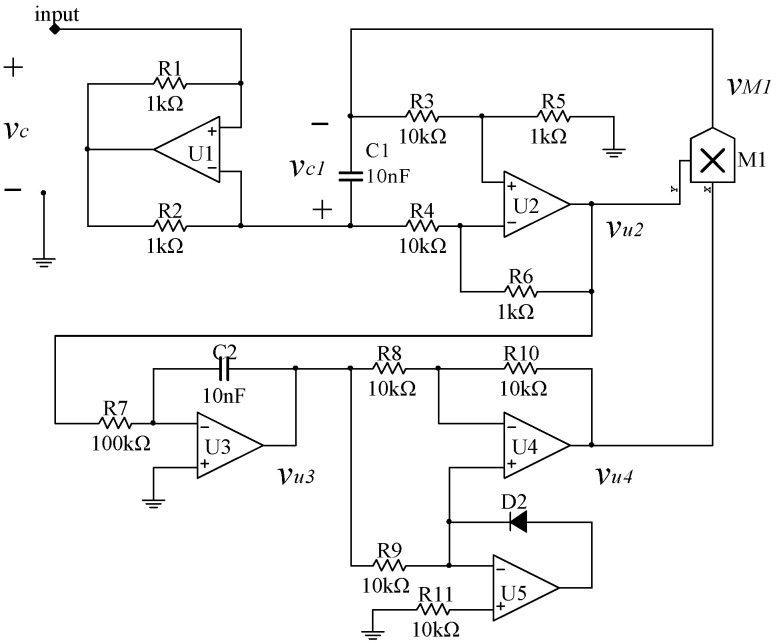
Equivalent circuit of the charge-controlled memcapacitor.

An emulating circuit is designed, as shown in Figure 1, to realize the above charge-controlled memcapacitor. In this circuit, it is assumed that all components are ideal without losses and the output limitations are set as ±15 V.

The circuit resistors are set as *R*_1_ = *R*_2_, *R*_3_ = *R*_4_, and *R*_5_ = *R*_6_. The operational amplifier *U*_1_ is used to reverse the sign of *i*, which is the current going into the floating terminal of the memcapacitor. Then, the charge *q* going through capacitor *C*_1_ can be calculated by integrating *i* in the time domain. The operational amplifier *U*_2_ constitutes a subtraction circuit, used to extract the voltage across the capacitor *C*_1_, with output voltage:

(3) $$v_{u2}\left(t\right)=-\frac{R_{6}}{R_{4}}v_{c1}=\frac{R_{6}}{R_{4}}\frac{q_{M}\left(t\right)}{C_{1}}$$

The operational amplifier *U*_3_ is an integral circuit and its output is:

(4) $$v_{u3}\left(t\right)=-\frac{1}{R_{7}C_{2}}\int v_{u2}\left(t\right)dt=-\frac{R_{6}}{C_{1}C_{2}R_{4}R_{7}}\int q_{M}\left(t\right)dt=-\frac{R_{6}}{C_{1}C_{2}R_{4}R_{7}}\sigma_{M}\left(t\right)$$

where *σ_M_*(*t*) is the integral of *q_M_*(*t*). 

Operational amplifiers *U*_4_ and *U*_5_ construct an absolute-valued circuit with output voltage:

(5) $$v_{u4}\left(t\right)=\left|v_{u3}\left(t\right)\right|=\frac{R_{6}}{C_{1}C_{2}R_{4}R_{7}}\left|\sigma_{M}\left(t\right)\right|$$

Now, based on Equations (3) and (5), the output voltage of the multiplier *M*_1_ can be calculated using:

(6) $$v_{M1}\left(t\right)=\frac{R_{6}^{2}}{C_{1}^{2}C_{2}R_{4}^{2}R_{7}}\left|\sigma_{M}\left(t\right)\right|q_{M}\left(t\right)$$

Then, the voltage across this grounded memcapacitor can be written as:

(7) $$v_{C}\left(t\right)=v_{C_{1}}\left(t\right)+v_{M1}\left(t\right)=\left(-\frac{1}{C_{1}}+\frac{R_{6}^{2}}{C_{1}^{2}C_{2}R_{4}^{2}R_{7}}\left|\sigma_{M}\left(t\right)\right|\right)q_{M}\left(t\right)$$

**Figure 2:**
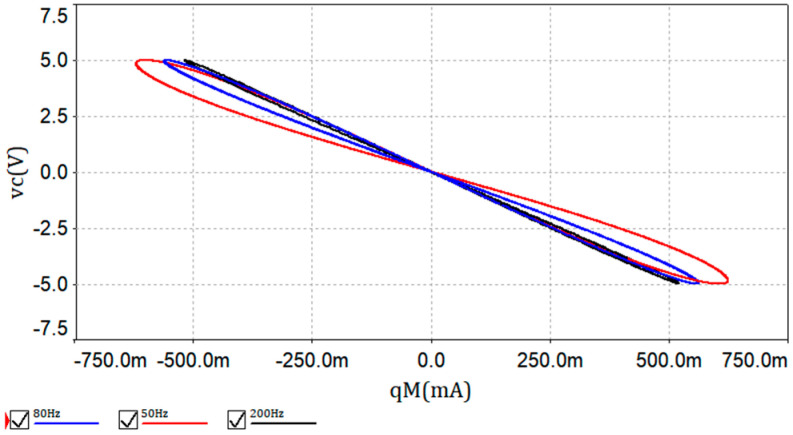
*q_M_–v_C_* characteristic curves of the memcapacitor.

If the memcapacitor-based emulator have voltage signals applied, the instantaneous responses are obtained as shown in Figure 2. The voltage signal is set as *v_input_* = 5sin(2*πf*), with *f* being tested at 50 Hz, 80 Hz, and 200 Hz. The simulations of pinched hysteresis loops, referred to *q_M_–v_C_* characteristics, can be got from Multisim 12 software, which are shown in Figure 2.

## 3. Memcapacitor-Based Chaotic Oscillator and Its Dynamics

### 3.1. Memcapacitor-Based Chaotic Oscillator

**Figure 3:**
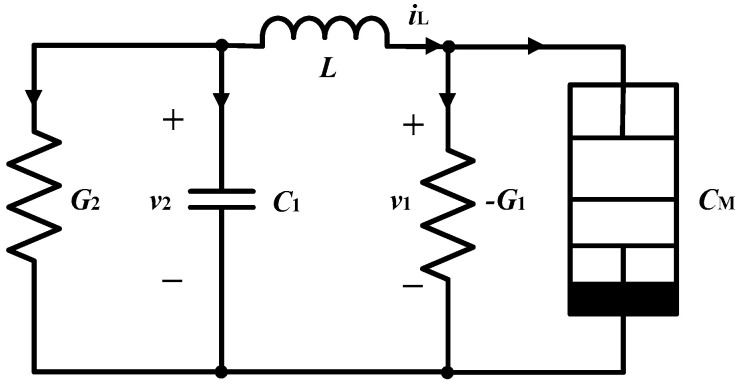
The memcapacitor-based chaotic oscillator.

Based on the memcapacitor model in Equation (7), a chaotic oscillator is designed as shown in Figure 3, which contains conductances *G*_1_ and *G*_2_, capacitor *C*_1_, inductor *L*, and memcapacitor *C_M_*.

**Figure 4:**
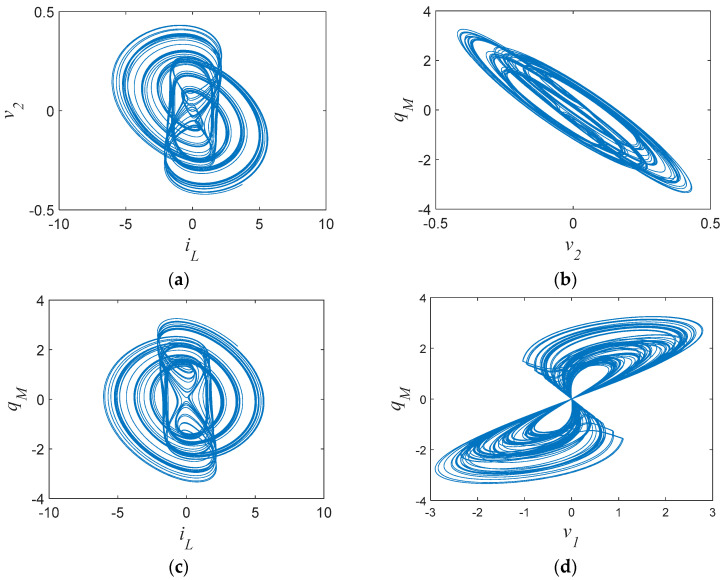
Chaotic attractors and chaotic *v*_1_–*q*_M_ hysteresis loops of the memcapacitor-based oscillator: (**a**–**c**) chaotic attractors; (**d**) chaotic *v*_1_–*q*_M_ hysteresis loops.

Taking the current *i_L_* through the inductor, the voltage *v*_2_ across the capacitor and the charge *q_M_* on the memcapacitor as state variables, a set of four first-order state equations can be obtained, as follows:

(8) $$\{\begin{array}{l} L\frac{di_{L}}{dt}=v_{2}-v_{1}\\ C_{1}\frac{dv_{2}}{dt}=-i_{L}-G_{2}v_{2}\\ \frac{dq_{M}}{dt}=i_{L}+G_{1}v_{1}\\ \frac{d\sigma_{M}}{dt}=q_{M} \end{array}$$

where *v*_1_ = (*a* + *b*|*σ_M_*(*t*)|)*q_M_*. Let the circuit parameters be chosen as shown in Table 1 with initial conditions (0.02, 0, 0, 0). Then, System (8) is chaotic with chaotic attractors as shown in Figure 4.

**Figure 5:**
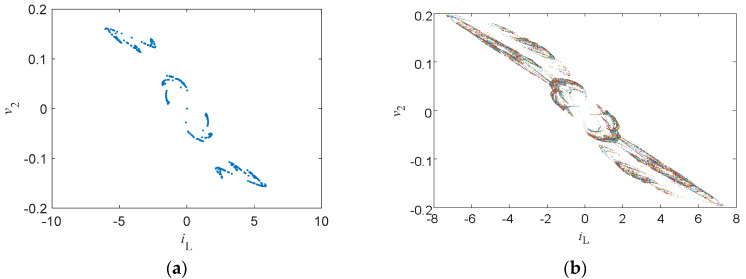
Poincaré maps on *z* = 0: (**a**) projection on the *x–y* plane, and (**b**) projection on the *x–y* plane with initial condition *v*_2_(0) varying in the range of (−0.08, 0.08).

The system Lyapunov exponents were *LE*_1_ = 0.0382, *LE*_2_ = 0.0067, *LE*_3_ = −0.0097, and *LE*_4_ = −0.2142. The Lyapunov dimension was *DL* = 3.1643. Figure 5a shows the Poincaré map on *z* = 0 and Figure 5b shows the Poincaré maps with initial condition *v*_2_(0) varying in the range of (−0.08, 0.08), showing that the system’s dynamic behaviors were affected by initial conditions. All the above results verified that System (8) is chaotic [23].

### 3.2. Equilibrium Points

The system equilibrium set can be calculated using *E* = {(*x*, *y*, *z*, *w*) | *i_L_ = v*_2_ = *q_M_* = 0, *σ_M_* = *c*} by solving the equations of ${\dot{i}}_{L}={\dot{v}}_{2}={\dot{q}}_{M}={\dot{\sigma }}_{M}=0$, in which there is a real constant parameter *c*.

The Jacobian matrix *J* at this equilibrium set *E* is:

(9) $$J=\left[\begin{array}{cccc} 0 & \frac{1}{L} & -\frac{a+b\left|c\right|}{L} & 0\\ -\frac{1}{C_{1}} & -\frac{G_{2}}{C_{1}} & 0 & 0\\ 1 & 0 & G_{1}\left(a+b\left|c\right|\right) & 0\\ 0 & 0 & 1 & 0 \end{array}\right]$$

The corresponding characteristic equation is:

(10) $$\lambda^{4}-\left(G_{1}a-G_{2}/C_{1}+G_{1}b\left|c\right|\right)\lambda^{3}+\frac{\left(C_{1}-G_{1}G_{2}L\right)\left(a+b\left|c\right|\right)+1}{C_{1}L}\lambda^{2}-\frac{\left(G_{1}-G_{2}\right)\left(a+b\left|c\right|\right)}{C_{1}L}\lambda$$

It is obvious that the characteristic equation has one zero value and three nonzero values. 

Let *a*_1_ = −(*G*_1_*a* − *G*_2_/*C*_1_ + G_1_*b|c|*), *a*_2_ = [(*C*_1_ − *G*_1_*G*_2_*L*)(*a* + *b*|*c*|) + 1]/*C*_1_*L*, *a*_3_ = −[(*G*_1_ − *G*_2_)(*a* + *b*|*c*|)]/*C*_1_*L.* Then, according to the Routh–Hurwitz condition, the system is stable if:

(11) $$\{\begin{array}{l} \Delta_{1}=a_{1}>0\\ \Delta_{2}=a_{1}a_{2}-a_{3}>0\\ \Delta_{1}=a_{3}\left(a_{1}a_{2}-a_{3}\right)>0 \end{array}$$

When the parameters are set as in Table 1, one can find that:

1.4002 < |*c*| *<* 2.7431 (12)

To make the equilibrium set *E* unstable, which is a necessary condition for the possible existence of chaos, the constant *c* should satisfy:

|*c*| *<* 1.4002  or  |*c*| *>* 2.7431 (13)

Equation (13) demonstrates that the dynamical behaviors of the chaotic circuit (8) were strongly dependent on the memcapacitor internal state variable *σ_M_*. For example, if the system parameters were set as in Table 1, with *c* = 1, then four eigenvalues at the equilibrium set *E* were obtained as:

λ_1_ = 0, λ_2_ = 0.54411, λ_3, 4_ = −0.45508 ± 0.26927*i* (14)

In this case, the equilibrium set *E* was unstable with one zero root, two complex conjugate roots with negative real parts, and one positive real root. Thus, a self-excited attractor could be generated via excitation from the unstable focal point in *E*.

### 3.3. Parameters Region

**Figure 6:**
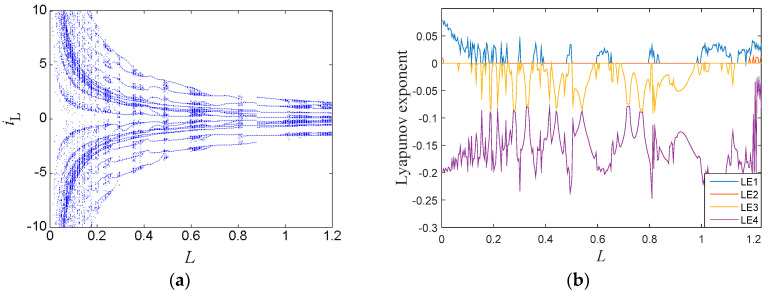
Dynamic characters with respect to *L*: (**a**) bifurcation diagram, and (**b**) lyapunov exponent spectrum.

When the inductor *L* increased gradually with other circuit parameters fixed as in Table 1, the bifurcation diagram of the state variable *i_L_* is shown in Figure 6a, where the orbits of the system started from chaotic behavior and then entered into periodic behavior via the reverse period-doubling bifurcation route. After that, the orbits returned to chaotic behavior through the forward period-doubling bifurcation route, then jumped into chaotic behavior, and finally approached infinity in the range of *L* > 1.25. The corresponding Lyapunov exponent spectra are presented in Figure 6b, where the maximum Lyapunov exponent was positive within chaotic regions and equalled zero within periodic regions. Several periodic windows can be observed in Figure 6b, which match well with that of the bifurcation diagram shown in Figure 6a.

**Figure 7:**
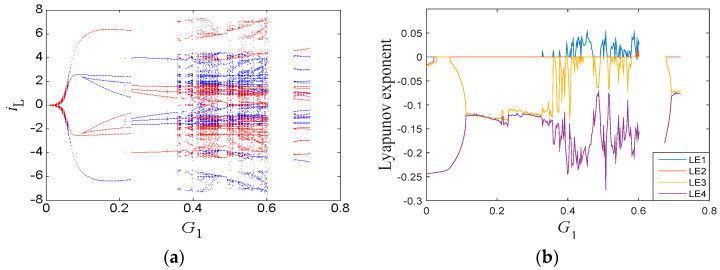
Dynamic characteristics due to *G*_1_: (**a**) bifurcation diagram, and (**b**) Lyapunov exponent spectrum.

The new system also had coexisting bifurcations. When the inductor *G*_1_ varied with other circuit parameters fixed as in Table 1, the bifurcation diagram of the state variable *i_L_* is shown in Figure 7a, where the red orbit started from initial conditions (0, −0.03, 0, 0), and the blue one starts from (0, 0.03, 0, 0). The symmetrically coexisting bifurcation orbits were generated by the symmetrical coexisting attractors, which are shown in Figure 9. The corresponding Lyapunov exponent spectrum is shown in Figure 7b. From the bifurcation diagram and the Lyapunov exponent spectrum, it can be seen that the system orbit started from a limit cycle and then turned into the chaotic state through period-doubling bifurcations in the range of (0, 0.6). When the parameter *G*_1_ > 0.6, the bifurcation diagram and the Lyapunov exponent spectrum had blank areas since the system was diverging with no solution. Between the two blank areas, the system stayed in a periodic state.

### 3.4. Similar Bifurcation Structures with Initial Conditions

**Figure 8:**
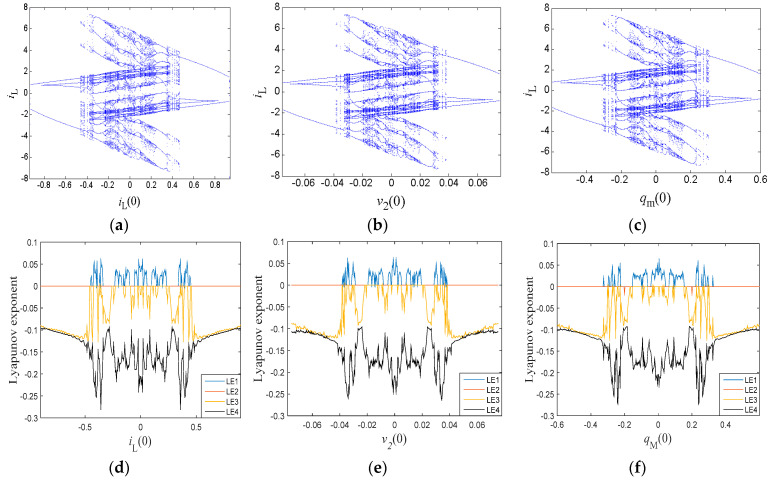
Dynamic characteristics: (**a**–**c**) bifurcation diagrams; and (**d**–**f**) Lyapunov exponent spectra.

Different bifurcation parameters usually lead to different bifurcation structures. However, there exist similar bifurcation structures with different initial conditions, which is rare compared with other chaotic systems. When we set the circuit parameters as given in Table 1, similar bifurcation diagrams with respect to *i_L_*(0), *v*_2_(0), and *q_M_*(0) were discovered, as presented in Figure 8. Although these diagrams depended on the various bifurcation parameters, it is remarkable that the bifurcation structures were almost the same and all symmetrical about the origin. As the bifurcation parameters increased gradually, the system orbit went to a chaotic status via period-doubling bifurcations. Then, the dynamics of the system settled down to periodic behaviors via reverse period-doubling bifurcations. The origin was the boundary of the two processes. The corresponding Lyapunov exponent spectra are given in Figure 8d–f, where the graphs also have similar shapes.

**Figure 9:**
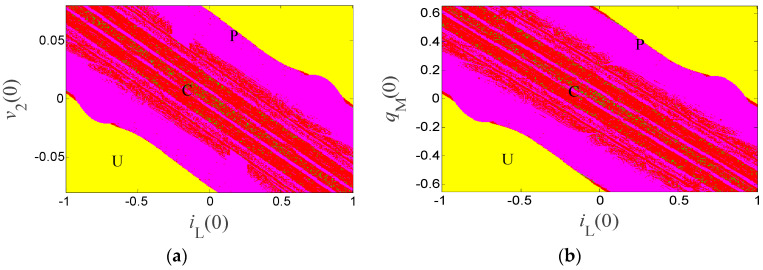
Dynamic maps of the system: (**a**) *i_L_*(0)*–v*_2_(0) plane, and (**b**) *i_L_*(0)*–q_M_*(0) plane.

Furthermore, the system’s dynamic maps are used to illustrate the similar initial-condition- triggered bifurcation structures, which are shown in Figure 9. These dynamic maps describe different dynamical regions with respect to the bifurcation parameters *i_L_*(0), *v*_2_(0), and *q_M_*(0), where the red areas indicate a chaotic field, the blue regions represent a periodic status, and yellow areas show unbounded zones. It is obvious the two dynamic maps own a similar distribution structure. Thus, we can conclude that the initial conditions *i_L_*(0), *v*_2_(0), and *q_M_*(0) had a similar dynamic influence for the presented system in the especial parameter spaces, which is not common in the other chaotic systems.

### 3.5. Extreme Multistability and Coexisting Attractors

Multistability is a common phenomenon in many nonlinear dynamical systems, corresponding to the coexistence of more than one stable attractor for the same set of system parameters [24]. When infinitely many attractors coexist for the same set of system parameters, multistability is referred to as extreme multistability [25]. The previously published literature have reported that the dynamical stability of memristive systems are heavily dependent on the initial conditions, which easily leads the system to generate multistability or even extreme multistability [26,27,28,29].

**Figure 10:**
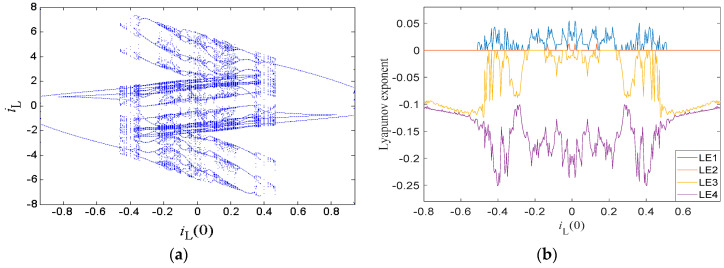
Dynamic characters due to *i_L_*(0): (**a**) bifurcation diagram, and (**b**) Lyapunov exponent spectrum.

One of the main features of extreme multistability is that the system track can present bifurcation without varying any system parameter. When the circuit parameters were set as in Table 1, with initial condition *i_L_*(0) varying, the resulting bifurcation diagram of the state variable *i_L_* is shown in Figure 10a, where the system presents extreme multistability. It is remarkable to see that the bifurcation diagram is symmetrical about the origin, which came from symmetrical coexisting attractors. As initial condition *i_L_*(0) increased gradually within the region of [−0.94, 0], the system orbit started from a limit cycle and turned into a chaotic state through period-doubling bifurcations, showing several periodic windows. Within the region of [0, 0.94], the system orbit settled down to periodic behavior from the chaotic state through reverse period-doubling bifurcations, which is the reverse evolutionary process of that in the region [−0.94, 0]. The corresponding Lyapunov exponent spectra are shown in Figure 10b, where the maximum Lyapunov exponents stay zero with limit cycles but were positive in chaotic states, consistent with the bifurcation diagram. 

**Figure 11:**
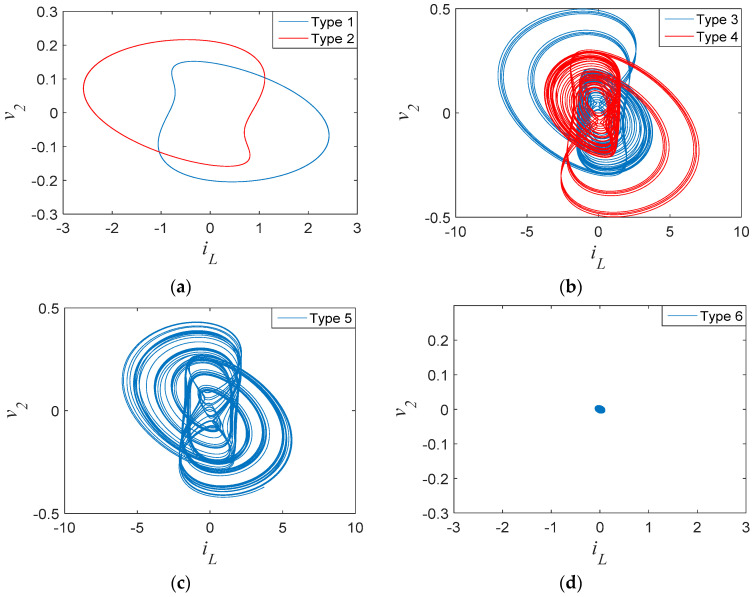
Phase portraits of coexisting attractors: (**a**) Type 1 and Type 2; (**b**) Type 3 and Type 4; (**c**) Type 5; (**d**) Type 6.

To show more details, various typical coexisting attractors are displayed in Figure 11. When the circuit parameters were set as in Table 1, with different initial conditions, the main coexisting regimes were symmetric pairs of limit cycles, chaotic attractors, and point attractors. Figure 11a shows limit cycles coexisting with attractors for initial conditions (0.02, ±0.06, 0, 0). Figure 11b,c shows coexisting chaotic attractors with initial values (0, ±0.04, 0, 0) and (0.02, 0, 0, 0), respectively. Figure 11d displays a point attractor with initial conditions (−1, 0.18, 0, 0). Since the system track presented bifurcations with initial conditions and had infinitely many coexisting attractors for the same set of system parameters, the system presented typical extreme multistability.

**Figure 12:**
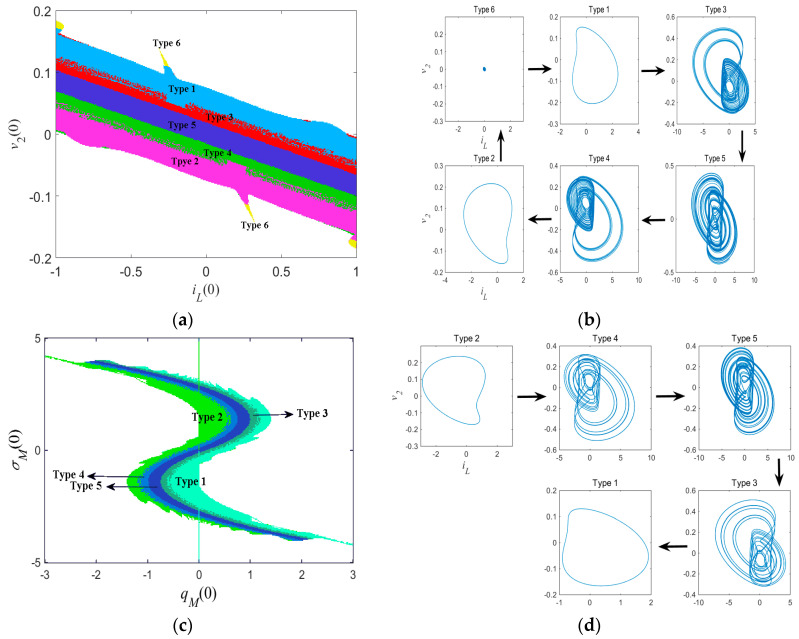
Basins of attraction of coexisting attractors in (**a**) the *i_L_*(0)*–v*_2_(0) plane and (**c**) the *q_M_*(0)*–σ*_M_(0) plane; the corresponding evolution process of coexisting attractors in (**b**) *i_L_*(0)*–v*_2_(0) plane and (**d**) the *q_M_*(0)*–σ*_M_(0) plane.

Basins of attraction can clearly display the distribution of different coexisting attractors. As shown in Figure 11, the system had six kinds of coexisting attractors. When initial conditions *i_L_*(0) and *v*_2_(0) were set as variables, with *q_M_*(0) = 0 and *σ_M_*(0) = 0, the corresponding basin of attraction is displayed in Figure 12a, where coexisting attractor distributions are painted with different colors. As shown in Figure 12a, the system was divergent with no attractor in most blank areas and generated a narrow distribution of each kind of the coexisting attractors in the middle areas. The evolution process of coexisting attractors is shown in Figure 12b, which contains a complete process of period-doubling bifurcations and reverse period-doubling bifurcations as *v*_2_(0) decreases. The system orbit started from a point attractor (Type 6) to a limit cycle (Type 1), and then turned to a chaotic attractor (Type 5) via period-doubling bifurcations (Type 3). As *v*_2_(0) decreased further, the chaotic attractor (Type 5) returned to a limit cycle (Type 2) via reverse period-doubling bifurcations (Type 4) and finally settled into a point attractor (Type 6).

Similarly, the basin of attraction with respect to initial conditions *q_M_*(0) and *σ_M_*(0) is displayed in Figure 12c, which is roughly symmetric about the origin and each attractor distribution appears in a narrow reverse ‘S’ shape. The corresponding evolution process of coexisting attractors is shown in Figure 12d.

## 4. Experimental Results

An analog electronic circuit was built to physically realize the above-presented chaotic oscillator to verify the basic dynamic behaviors of the new system. 

**Figure 13:**
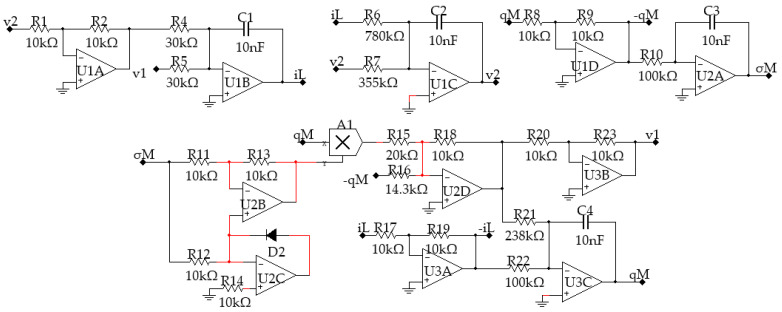
The equivalent circuit of the new system.

**Figure 14:**
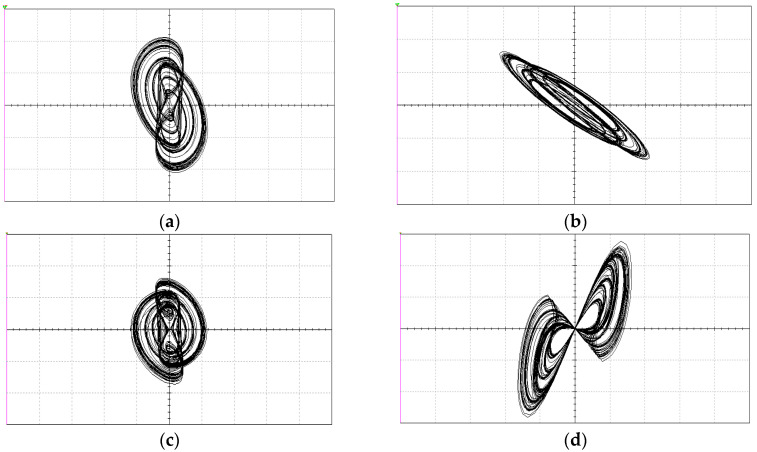
Chaotic attractors observed via Multisim simulations: (**a**–**c**) chaotic attractors; and (**d**) chaotic *v*_1_–*q*_M_ hysteresis loops.

Since the inductor and the capacitor in the system were not standard, which made the circuit difficult to design and implement, an equivalent circuit was designed to realize the system. The circuit schematic of the experimental circuit is shown in Figure 13, and the corresponding chaotic attractors obtained by Multisim simulation are displayed in Figure 14. 

**Figure 15:**
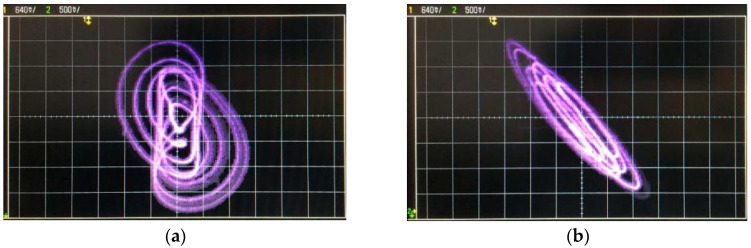

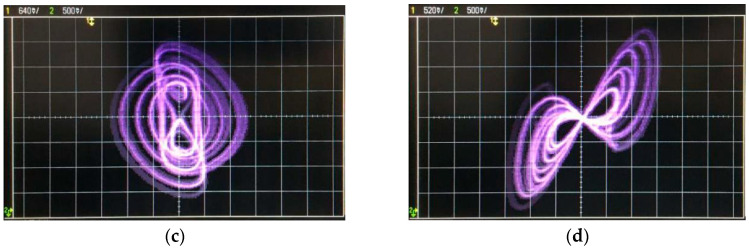
Chaotic attractors observed from the oscilloscope in the experiment: (**a**–**c**) chaotic attractors; and (**d**) chaotic *v*_1_–*q*_M_ hysteresis loops.

In practical experiments, since there are tolerances in resistors and capacitors, it is necessary to adjust the actual resistance values in the analog circuit, which will lead to some deviations between experimental results and the simulation ones. The experimental results shown on the oscilloscope are depicted in Figure 15. The chip AD633JN was chosen as the analog multiplier and LF347N as the operational amplifier with reference voltages of ±15 V. It is clear that the dynamical behaviors observed from the experimental circuit were generally similar with those displayed via numerical simulations.

## 5. Conclusions

In this paper, a new memcapacitor model and a novel memcapacitor-based chaotic circuit are presented. The system extreme multistability was analyzed, including bifurcation diagrams, Lyapunov spectra, coexisting attractors, coexisting bifurcations, and basins of attraction of various attractors. Moreover, the new memcapacitor-based system was realized using an experimental circuit, which agreed well with the numerical simulations and verified the theoretical analysis results. Due to the rich and unusual complex dynamical characteristics of the proposed memcapacitor system, it was deemed that it would find some novel and non-traditional applications in engineering and technology in the future. In our future works, we will continue to try to build physical memcapacitors and explore special dynamics in memcapacitive circuits. 

## Figures and Tables

**Table 1:** Circuit parameters for simulations and experiments.

Parameters	Meanings	Values
*L*	Inductance	0.3 mH
*C*_1_	Capacitor	7.8 nF
*G*_1_	Conductance	0.42 mS
*G*_2_	Conductance	2.2 mS
*a*	Variable	−0.7
*b*	Variable	0.5

## References

[1] Ventra M.D., Pershin Y.V., Chua L.O. (2009). Circuit elements with memory: Memristors, memcapacitors and meminductors. Proc. IEEE.

[2] Strukov D.B., Snider G.S., Stewart D.R., Williams R.S. (2008). The missing memristor found. Nature.

[3] Martinez-Rincon J., Pershin Y.V. (2011). Bistable non-volatile elastic membrane memcapacitor exhibiting chaotic behavior. IEEE Trans. Electron Dev..

[4] Lai Q., Zhang L., Li Z., Stickle W.F., Williams R.S., Chen Y. (2009). Analog memory capacitor based on field-configurable ion-doped polymers. Appl. Phys. Lett..

[5] Guo M., Xue Y.B., Gao Z.H., Zhang Y.M., Dou G., Li Y.X. (2017). Dynamic analysis of a physical SBT memristor-based chaotic circuit. Int. J. Bifurc. Chaos.

[6] Wang S.B., Wang X.Y., Zhou Y.F. (2015). A memristor-based complex lorenz system and its modified projective synchronization. Entropy.

[7] Wang S.B., Wang X.Y., Zhou Y.F., Han B. (2016). A memristor-based hyperchaotic complex Lu system and its adaptive complex generalized synchronization. Entropy.

[8] Xi H.L., Li Y.X., Huang X. (2014). Generation and nonlinear dynamical analyses of fractional-order memristor-based Lorenz systems. Entropy.

[9] Chen M., Yu J.J., Yu Q., Li C.D., Bao B.C. (2014). A memristive diode bridge-based canonical chua’s circuit. Entropy.

[10] Martinez-Rincon J., Di Ventra M., Pershin Y.V. (2010). Solid-state memcapacitive system with negative and diverging capacitance. Phys. Rev. B.

[11] Biolek D., Biolek Z., Biolkova V. (2011). Behavioral modeling of memcapacitor. Radioengineering.

[12] Biolek D., Biolek Z., Biolkova V. (2010). Spice modeling of memcapacitor. Electron. Lett..

[13] Fouda M.E., Radwan A.G. (2014). Memcapacitor response under step and sinusoidal voltage excitations. Microelectron. J..

[14] Biolek D., Biolkova V. (2010). Mutator for transforming memristor into memcapacitor. Electron. Lett..

[15] Fouda M.E., Radwan A.G. (2015). Boundary dynamics of memcapacitor in voltage-excited circuits and relaxation oscillators. Circuits Syst. Signal Process..

[16] Wang G.Y., Shi C.B., Wang X.W., Yuan F. (2017). Coexisting oscillation and extreme multistability for a memcapacitor-based circuit. Math. Probl. Eng..

[17] Yu D.S., Zhou Z., Iu H.H.C., Fernando T., Hu Y.H. (2016). A coupled memcapacitor emulator-based relaxation oscillator. IEEE Trans. Circuits Syst. II-Express Briefs.

[18] Yuan F., Wang G.Y., Shen Y.R., Wang X.Y. (2016). Coexisting attractors in a memcapacitor-based chaotic oscillator. Nonlinear Dyn..

[19] Wang G.Y., Zang S.C., Wang X.Y., Yuan F., Iu H.H.C. (2017). Memcapacitor model and its application in chaotic oscillator with memristor. Chaos.

[20] Yuan F., Wang G.Y., Wang X.W. (2017). Chaotic oscillator containing memcapacitor and meminductor and its dimensionality reduction analysis. Chaos.

[21] Rajagopal K., Akgul A., Jafari S., Aricioglu B. (2018). A chaotic memcapacitor oscillator with two unstable equilibriums and its fractional form with engineering applications. Nonlinear Dyn..

[22] Yamaletdinov R.D., Ivakhnenko O.V., Sedelnikova O.V., Shevchenko S.N., Pershin Y.V. (2018). Snap-through transition of buckled graphene membranes for memcapacitor applications. Sci. Rep..

[23] Lassoued A., Boubaker O. (2017). On new chaotic and hyperchaotic systems: A literature survey. Nonlinear Anal. Model. Control.

[24] Feudel U. (2008). Complex dynamics in multistable systems. Int. J. Bifurc. Chaos.

[25] Ngonghala C., Feudel U., Showalter K. (2011). Extreme multistability in a chemical model system. Phys. Rev. E.

[26] Chen M., Sun M.X., Bao B.C., Wu H.G., Xu Q., Wang J. (2018). Controlling extreme multistability of memristor emulator-based dynamical circuit in flux-charge domain. Nonlinear Dyn..

[27] Bao H., Jiang T., Chu K.B., Chen M., Xu Q., Bao B.C. (2018). Memristor-based canonical Chua’s circuit: Extreme multistability in voltage-current domain and its controllability in flux-charge domain. Complexity.

[28] Boubaker O., Jafari S. (2018). Recent Advances in Chaotic Systems and Synchronization: From Theory to Real World Applications.

[29] Rajagopal K., Jafari S., Karthikeyan A., Srinivasan A., Ayele B. (2018). Hyperchaotic memcapacitor oscillator with infinite equilibria and coexisting attractors. Circuits Syst. Signal Process..

